# Analysis of genomic rearrangements, horizontal gene transfer and role of plasmids in the evolution of industrial important *Thermus* species

**DOI:** 10.1186/1471-2164-15-813

**Published:** 2014-09-25

**Authors:** Benjamin Kumwenda, Derek Litthauer, Oleg Reva

**Affiliations:** Department of Biochemistry, Bioinformatics and Computational Biology Unit, University of Pretoria, Pretoria, South Africa; Department of Microbial Biochemical and Food Biotechnology, University of Free State, Bloemfontein, South Africa

**Keywords:** Rearrangements, Metabolic networks, Clustering, Genomic island, Thermophile

## Abstract

**Background:**

Bacteria of genus *Thermus* inhabit both man-made and natural thermal environments. Several *Thermus* species have shown biotechnological potential such as reduction of heavy metals which is essential for eradication of heavy metal pollution; removing of organic contaminants in water; opening clogged pipes, controlling global warming among many others. Enzymes from thermophilic bacteria have exhibited higher activity and stability than synthetic or enzymes from mesophilic organisms.

**Results:**

Using *Meiothermus silvanus* DSM 9946 as a reference genome, high level of coordinated rearrangements has been observed in extremely thermophilic *Thermus* that may imply existence of yet unknown evolutionary forces controlling adaptive re-organization of whole genomes of thermo-extremophiles. However, no remarkable differences were observed across species on distribution of functionally related genes on the chromosome suggesting constraints imposed by metabolic networks. The metabolic network exhibit evolutionary pressures similar to levels of rearrangements as measured by the cross-clustering index. Using stratigraphic analysis of donor-recipient, intensive gene exchanges were observed from *Meiothermus* species and some unknown sources to *Thermus* species confirming a well established DNA uptake mechanism as previously proposed.

**Conclusion:**

Global genome rearrangements were found to play an important role in the evolution of *Thermus* bacteria at both genomic and metabolic network levels. Relatively higher level of rearrangements was observed in extremely thermophilic *Thermus* strains in comparison to the thermo-tolerant *Thermus scotoductus*. Rearrangements did not significantly disrupt operons and functionally related genes. *Thermus* species appeared to have a developed capability for acquiring DNA through horizontal gene transfer as shown by the donor-recipient stratigraphic analysis.

**Electronic supplementary material:**

The online version of this article (doi:10.1186/1471-2164-15-813) contains supplementary material, which is available to authorized users.

## Background

Bacteria of the genus *Thermus* inhabit both natural and man-made thermal environments such as hot springs, deep mines, compost manure, sewage sludge and domestic hot water [1, 2]. *Thermus* bacteria are of major interest because of their industrially important thermostable enzymes; their ability to reduce heavy metals and switch to anaerobic respiration under oxygen deprived conditions. Enzymes from thermophilic organisms have shown higher activity and stability than mesophilic or synthetic enzymes counterparts currently been used in industry for production of food, detergents, drugs and paper [3]. *Thermus scotoductus* SA-01 in particular, has been found to reduce heavy metals such Fe(III), Cr(VI), Mn(IV), U(VI) and Co(III) [4, 5]. Reduction of Fe(III) and Mn(VI) can be applied in biotechnology for eradicating heavy metal pollution; controlling global warming; removing organic contaminants in ground water; fluxing phosphates and other contaminants from water supplies; and also for clearing clogged wells among many other uses [6]. Fe(III) reduction under anaerobic conditions in swampy areas during flooding diverts electrons away from methane producers thereby reducing global methane fluxes into the atmosphere consequently lowering global warming. Anaerobic respiration is advantageous in bio-fuel production as temperature rises and oxygen depletes due to decomposition of biomass. Cr(IV) is cacogenic; hence its reduction eliminates toxicity in food and air for human health [7].

Mutations and natural selection have been known to be dominant drivers of microbial evolution until the observation of abrupt changes in traits of an organism such as acquisition of pathogenicity or drug resistance that could not be explained by predominantly known ordinary mechanisms in time. Since then, horizontal gene transfer through which organism acquire foreign DNA to incorporate into their genomes through conjugation, transduction and transformation has been used to explain the ‘quantum leaps’ in traits of organisms that defy neo-Darwinian theories. Genome plasticity due to natural transformations is considered as a major survival technique for *Thermus* species in extreme temperature environments [8]. It is known that co-expressed and functionally related genes in bacteria are grouped into operons or co-localised on the chromosome creating a network of reusable functional blocks [9, 10]. However, introduction of new genes by horizontal gene transfer and genome rearrangements affect the order of genes and may disrupt operon structure that consequently may lead to metabolic network re-organisation. Genomic recombinations are involved in evolution and speciation of organisms in addition to other mechanisms such as mutations, natural selection and horizontal gene transfer [11]. What triggers rearrangements and determine their locations on the chromosome remains unknown. The extent to which thermal environments affect genome rearrangements on the chromosome or exert evolutionary pressure on the metabolic network is also not clear. Both the retrograde and patchwork theories attempt to explain the evolution of metabolic networks based on gene and operon duplication linking distribution of genes on the chromosome which may be affected by rearrangements and consequently on the structure of the metabolic network [12]. Comparative analysis of genes and genomes in Archea, Bacteria and Eukarya has revealed that different forces and molecular mechanism might have shaped genomes leading to new metabolic capabilities essential for adaptation and survival [13]. Schwarzenlander *et al.* [8] and Friedrich *et al.* [14] observed high levels of natural transformation and identified a DNA uptake system encoded by 12 competent genes which code for pilin like proteins similar to type IV pilus biogenesis proteins. Eleven of which were identified and implicated in binding naked DNA from the environment, transporting it through the cell wall, outer and inner membranes into the cytoplasm. In *T. thermophilus* HB27, DNA binding is achieved by *pilQ*, transported through the outer cell membrane by *comEA*, *pilF* and *pilA4*, through the thick cell wall layers and inner membrane by *pilM*, *pilN*, *pilO*, *pilA13* and *comEC*. Whilst prior work by Gouder *et al.* [15] performed a comprehensive analysis of genomic islands possibly acquired through natural transformations, and their functional contribution in *Thermus* species, this work investigated movement of genomic islands and the ability for *Thermus* species to acquire external DNA.

**Figure 1:**
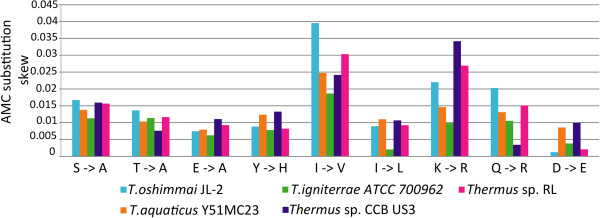
**Skewed amino acid substitutions in proteins of** ***Thermus*** **extreme thermophiles compared to their orthologs in the thermo-tolerant** ***T. scotoductus*** **SA-01.** Values of skewness were calculated as the difference between the number of substitutions of an amino acid A1 in *T. scotoductus* with A2 in ortholog proteins and the number of reverse substitutions normalized by numbers of matches of amino acids A1 and A2 in both genomes. See more details in Kumwenda *et al*. [16].

In a previously published work [16] we discovered several general trends in amino acid substitutions consistent with differences in thermostability between the thermo-tolerant *Thermus scotoductus* SA-01 (inhabits environments with temperatures between 60 to 65°C) and the extreme thermophiles *Thermus thermophilus* HB8 and HB27 (growth temperatures ranges of 65 to 85°C). During the year after this publication, genome sequences of many other extremely thermophilic species of the genus *Thermus* have become available: *T. aquaticus* Y51MC23, *Thermus* sp. RL [17], *T. igniterrae* ATCC 700962, *T. oshimai* JL-2 [18], *Thermus* sp. CCB US3 UF1 [19] and several others. Despite taxonomic diversity of these species that will be discussed below, we identified the same trends of accumulation of specific amino acids in proteins of extreme thermophiles compared to their orthologs in *T. scotoductus* (Figure 1) that we discovered before in a few *T. thermophilus* strains [16]. Thermostable proteins of *Thermus* organisms were characterized with a greater number of alanine residues accumulated by replacing serine, threonine and glutamate with this amino acid; frequent substitutions of isoleucine to leucine and valine; accumulation of arginine by substituting lysine and glutamine; and a decreased frequency of aspartate substituted by glutamate.

Against this background, we theorized that there may be several general trends in the whole genome adaptation to the high temperature environment in *Thermus* extreme thermophiles. Therefore we investigated the extent to which *Thermus* genomes have been shuffled and disintegrated due to rearrangements; how genome rearrangements affected functionally related genes and consequently exerted evolutionary pressure on the metabolic network. To investigate these questions, we performed a comparative genome analysis of *Thermus scotoductus* SA-01 (GenBank: NC_014974), *Thermus thermophilus* HB8 (NC_006461) and *Thermus thermophilus* HB27 (NC_005835). In some cases the comparative analysis was performed against *Meiothermus silvanus* DSM 9946 (CP002042), *Meiothermus ruber* (NC_013946), *T. aquaticus* Y51MC23 (ABVK02000000), *Thermus* sp. RL (AIJQ00000000), *T. igniterrae* ATCC 700962 (AQWU01000001), *T. oshimai* JL-2 (NC_019386-88), *Thermus* sp. CCB US3 UF1 (NC_017287 and NC_016634); and also against mesophilic *Escherichia coli* K-12 (NC_000913) and *Bacillus subtilis* (NC_000964). Furthermore, relative age and the movement of genomic islands among bacteria genomes have been investigated with the aim of analysing competence levels of *Thermus* species.

## Methods

### Identification of horizontally transferred genomic islands and single genes

Genomic islands in bacterial genomes were predicted by the SeqWord Genome Browser tool [20] and its semi-automatic realization SeqWord Sniffer [21], which are available at the SeqWord project website [22]. To identify genes which can be acquired by DNA uptake and homologous replacement, gene trees were designed for all sets of orthologous genes and their topologies were compared against the consensus species tree. This analysis was implemented using an in-house Python script that utilizes PHYLIP package command line programs PROTDIST, NEIGHBOR and TREEDIST [23].

### Identification of orthologous genes

Pairs of genes in two genomes were considered as orthologs if they reciprocally returned the best BLASTp hits in queries of predicted protein sequences of one genome against the whole set of proteins of another genome by using local implementations of BLAST and FORMATDB algorithms from NCBI [24] and an in-house BioPython based script for pipelining and output analysis. On the next step, MUSCLE alignment [25] was used to filter out false positive BLASTp predictions when the alignment covered less than 70% of the protein sequences in a pair of predicted orthologs. Resulting alignment files were used in designing gene trees as described above, but prior to phylogenetic analysis every alignment file was edited by the Gblocks program to remove ambiguous blocks [26].

### Co-localization of functionally related genes on the chromosome

Distances between genes on the chromosome were assigned to four distance categories: 0–1,000; 1,001-10,000; 10,001-100,000; 100,001-1,000,000. A biological meaning of these distance categories is that the first category apply to genes belonging to the same operon, the second category was based on the average gene length and the subsequent categories incremented by an order of these magnitudes [27]. To determine expected distribution of genes on the chromosome, average distances were measured between pairs of genes, which were randomly selected. Expected values were predicted based on a hypothesis of random distribution of genes on the chromosome. Observed values were calculated by computing distances between all functionally related genes in a genome in a pair wise manner and then allocating them to their respective distance categories. These were enzymes which acted on the same metabolites in the same metabolic pathways as predicted by the Pathway Tools software [28]. Co-localization of functionally related genes was estimated as a logarithm of the ratio of observed over expected frequencies of gene pairs calculated for each distance category normalised by genome length to eliminate bias.

### Genome Rearrangements and Phylogenetic analysis

Genome rearrangement events (relocations) were detected by finding discontinuities in gene syntenies in bacterial chromosomes aligned by Mauve 2.3.1 [29]. Gene orthology was determined as previously discussed. For ortholog sequence alignment and phylogenetic inference, programs Muscle [25], Gblocks [26], neighbor.exe [23], Maximum Likelihood algorithms implemented in PHYLIP [23] and Mega5 [30] and SplitsTree for phylogenetic network analysis [31] were used.

### Analysis of metabolic networks and metabolic clustering

The Pathways Tools software [28] was used to reconstruct metabolic pathways and operons based on genome annotations. The cross-clustering coefficients were calculated based on the method described by Spirin et al. [10]. Two genes encoding enzymes that use the same chemical compound either as a substrate or product were considered as ‘functional neighbors’, or in other words, having a metabolic edge. To simplify the network and avoid creation of unimportant or redundant links, abundant chemicals (such as water, ATP, enzyme co-factors, etc.) with more than 10 links between genes were discarded from consideration. Given that there are metabolic edges from gene *i* to genes *j* and *k*, the cross-clustering coefficient of the node *i* is the probability of having a genomic edge between its neighbors *j* and *k.* Nodes *j* and *k* have a genomic edge between them if they are co-localized in the same operon of the chromosomal DNA or the distance between them is not greater than an average length of operons. In this study, the average length of operons was estimated at 10,000 bases. The genome-wide cross-clustering coefficient is calculated as an average for all nodes *i* for the entire metabolic network. To avoid miss-associations or over-associations the analysis was limited to well annotated genes which participate in 38 common pathways predicted in *Thermus scotoductus* SA-01, *Thermus thermophilus* strains HB8 and HB27, *E. coli* and *Bacillus subtilis* strain 168.

## Results and Discussion

Bacterial evolution at genomic level involves accumulation of mutations, genome rearrangements and horizontal gene transfer. The contribution of all these different and independent evolutionary events towards speciation and adaptation of thermophilic bacteria of genus *Thermus* were analysed. *Thermus* bacteria is of industrial interest due to their ability to withstand extreme abiotic stresses including the high temperature and high-energy irradiation [8]; and also because of their role in decontamination of the environmental pollutions [32, 33] and ability to synthesize thermostable enzymes for industrial application [5].

### Identification of orthologous genes

**Figure 2:**
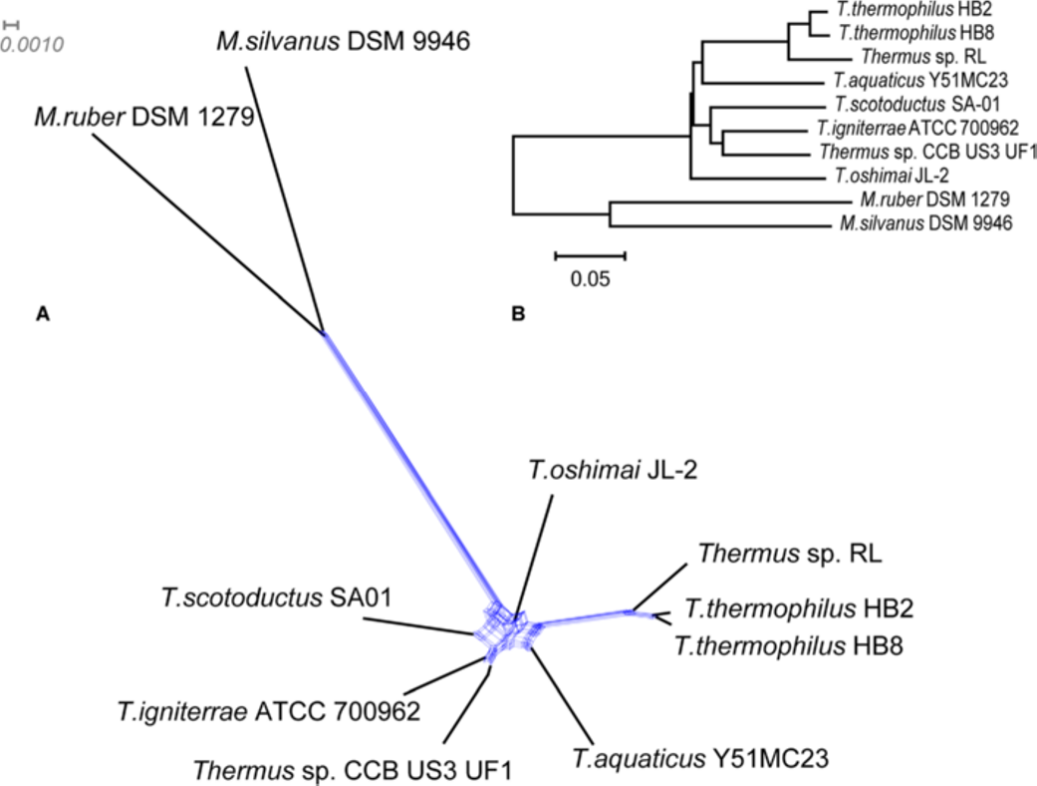
**Phylogenetic relationships between studied organisms. A)** Reticulation network created based on an analysis of individual gene trees by SplitsTree. Blue lines indicate possible gene exchange events between species. **B)** Neighbour-Joining phylogenetic tree based on a super-alignment of 1,441 orthologous proteins.

To identify orthologous genes for investigating possible gene exchanges among various bacteria species, a BLASTp search was done in a pair-wise manner for all coding sequences of 10 sampled genomes: *Thermus thermophilus* HB8 and HB27, *T. scotoductus* SA-01, *T. aquaticus* Y51MC23, *T. igniterrae* ATCC 700962, *T. oshimai* JL-2, *Thermus* sp. RL, *Thermus* sp. CCB US3 UF1, *Meiothermus silvanus* DSM 9946 and *Meiothermus ruber* DSM 1279. In total 1,441 groups of orthologous protein shared by 10 studied genomes were found. All these sequences were aligned by MUSCLE and individual gene trees for each alignment where created by the Neighbour-Joining (NJ) algorithm using PHYLIP executable files and the whole set of trees was analysed by SplitsTree to re-build a reticulation network (Figure 2A). Another approach of phylogenetic reconstruction was concatenating all alignments into a super-alignment of the total length of 390.024 amino acid residues. The resulted phylogenetic tree designed by the program MEGA 5 by using the Neighbour-Joining approach is shown in Figure 2B. It was concluded that extremely thermophilic strains of *Thermus* belonged to rather versatile species and very likely evolved independently from a thermo-tolerant ancestor. Phylogenetic network analysis revealed a number of possible reticulation events between these species especially in lineages *Meiothermus* and *T. thermophilus*. The phylogenetic network did not show directions of gene exchange (reticulation) events, i.e. an acquisition of a gene by a *Thermus* organism from the *Meiothermus* lineage would create a split in the phylogenetic network in the same way as a backward gene exchange. In the following section we tried to predict the directions of gene exchange by analysing topologies of individual gene trees.

### Genome Rearrangements

**Figure 3:**
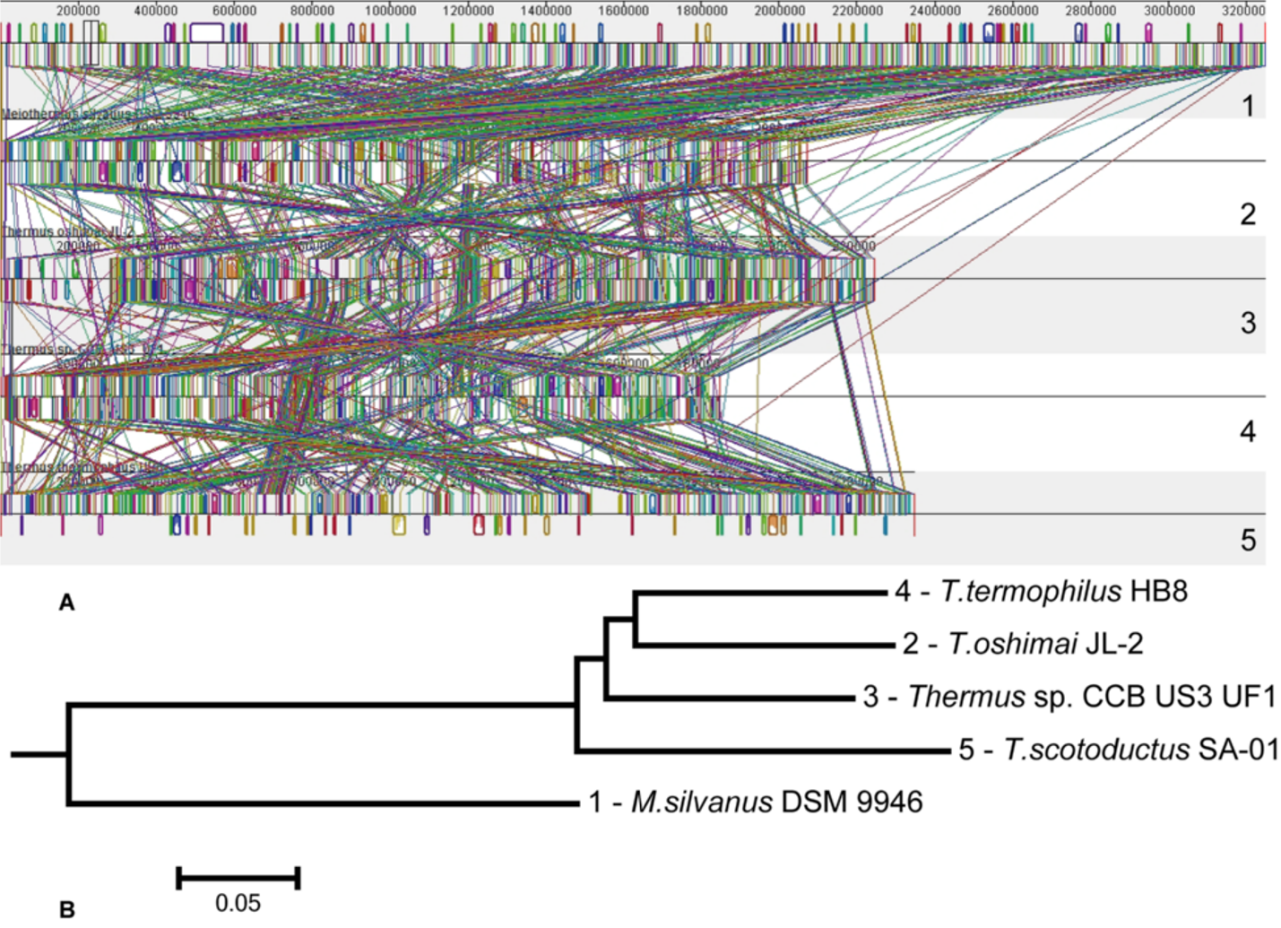
**Genome rearrangements in whole genome sequenced** ***Thermus*** **species. A)** Whole chromosome alignment by Mauve progressive alignment algorithm **B)** Clustering of aligned chromosomes by number and distribution of chromosomal rearrangements. *M. silvanus* DSM 9946 was used as the reference genome.

Bacteria of the genus *Thermus* are characterized with remarkably higher levels of genome rearrangements [15]. DNA fragments of different length were constantly mobile and moving to new locations on the chromosomes of these organisms. Based on the analysis of the phylogenetic tree in Figure 2, *Meiothermus silvanus* DSM 9946 was identified as a suitable reference genome to investigate rearrangements in *Thermus* organisms, as it was at an approximately equal evolutionary distance from the target genomes. Alignment of sequences of whole chromosomes was performed by the program Mauve only for 5 organisms of which complete genome sequences were finished (Figure 3A). The progressive alignment algorithm implemented in Mauve allows also building a phylogenetic tree based on analysis of genome rearrangements (Figure 3B). A great number of rearrangements were noted and it was an interesting observation that the extreme thermophiles *T. thermophilus*, *T. oshimai* and *Thermus* sp. CCB US3 UF1 were clustered together and apart from the thermo-tolerant *T. scotoductus* (Figure 3B) despite their taxonomic diversity (Figure 2B). More rearrangements were observed in extreme thermophiles as compared to *T. scotoductus* SA-01 (note bigger synteny blocks in the chromosome of *T. scotoductus* in Figure 3A), however this difference was not statistically reliable. While there is no biological evidence to back up rearrangements as an adaptation mechanism in thermophilic organisms, it may be possible that some unknown adaptation mechanism to thermal environments triggers them. In the further study we focused on comparison of *M. silvanus* DSM 9946, *T. scotoductus* SA-01, *T. thermophilus* HB8 and HB27 as representatives of thermo-tolerant and extremely thermophilic organisms.

**Figure 4:**
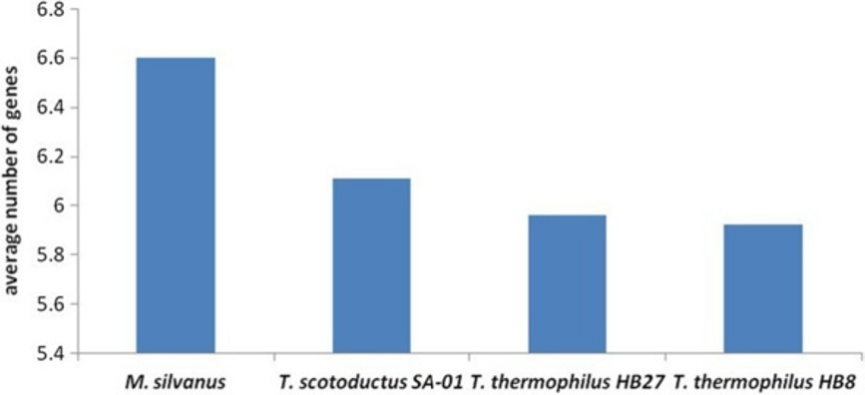
**Average numbers of genes per operons calculated for different genomes.**

A comparison of average lengths of operons (average number of genes) predicted by Pathway Tools software showed that *M. silvanus* DSM 9946 operons were longer as compared to those of SA-01, HB8 and HB27 (Figure 4). However, the pair-wise parametric *t-test* and non-parametric *Wilcoxon t-test* showed that differences in operon length were not statistically significant at 95% level of confidence. To some extent, this observation demonstrated some level of disintegration of operons in *Thermus* genomes resulting from possible frequent rearrangements, but at much lower level than it may be expected reasoning from the observed total number of rearrangements. *Thermus thermophilus* is adopted to survive extreme temperatures and it may be hypothesized that higher temperature environment can be associated with higher levels of rearrangements, or contrary, that the adaptation to higher temperature environments results in disintegration of operons and consequent higher levels of genome rearrangements. However, both hypotheses need further investigation.

**Figure 5:**
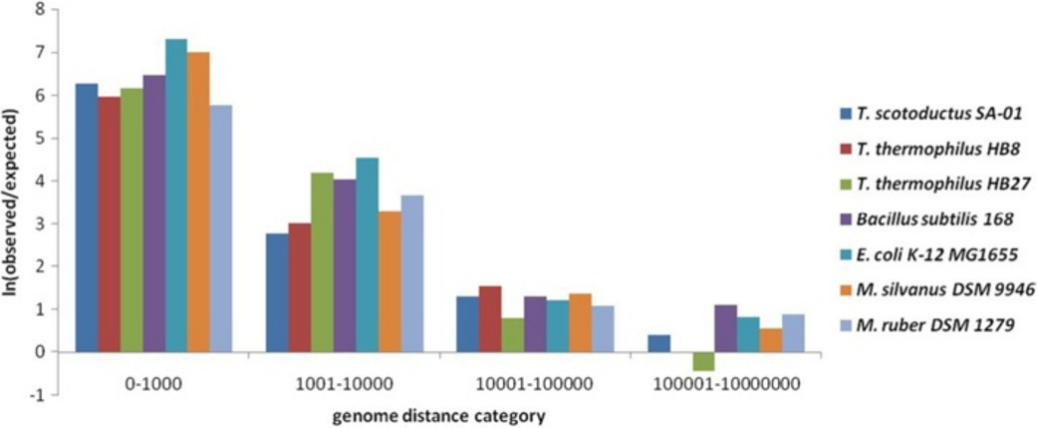
**Distribution of functionally related genes through various distance categories.**

**Figure 6:**
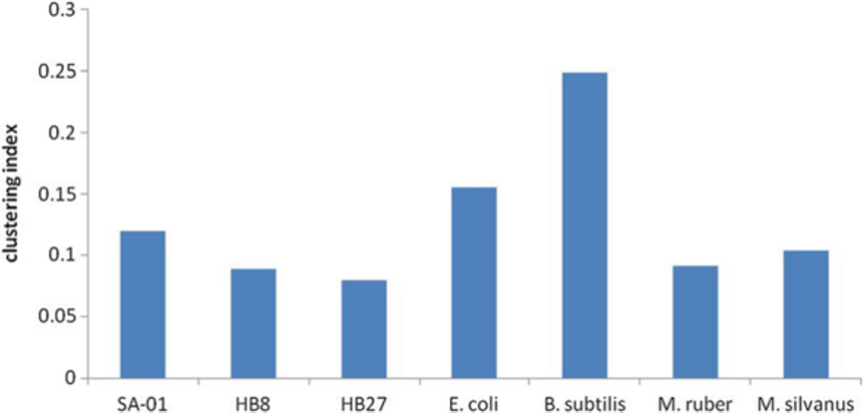
**Cross-clustering coefficients calculated for metabolic networks of different organisms.**

It was reported for most bacteria that genes encoding enzymes, which are functionally related and involved in the same metabolic pathways, are often co-localised on the chromosome [10]. It was interesting to investigate how the permanent shuffling of genomic blocks affected the distribution of functionally related genes. In this study, the general genome organization was investigated in thermophilic *T. scotoductus* SA-01 and *T. thermophilus* (HB8 and HB27); moderate thermophiles (*M. silvanus* DSM 9946 and *M. ruber* DSM 1279) and mesophilic bacteria (*Escherichia coli* K12 and *Bacillus subtilis* 168). Metabolic pathways were predicted by the Pathways Tools software [28]. Figure 5 shows logarithms of observed over expected pairs of functionally linked genes in various genomic distance categories. It was observed in all studied genomes that genes, which shared the same pathways and metabolites, in all studied organisms were more co-localized on the chromosome contrary to the expected hypothesis of random distribution of genes. There was no significant difference in the distribution of functionally related genes between thermophilic and mesophilic organisms. To estimate the differences in evolutionary pressures on metabolic networks as affected by genome rearrangements, cross-clustering coefficients were calculated (Figure 6). *B. subtilis* and *E. coli* showed significantly higher level of clustering of functionally related genes than *Thermus* and *Meiothermus* species; however it remained unclear whether this dispersed distribution of genes in latter genomes was a result of adaptation to harsher environment or just a neutral biological property of these organisms. The level of metabolic network clustering in genomes of extreme thermophiles *T. thermophilus* HB8 and HB27 was much lower in support of the hypothesis of thermal adaptation. However, the observed differences between cross-clustering coefficients of *Thermus* and *Meiothermus* species were statistically insignificant.

Breakpoints of global genome rearrangements were distributed randomly throughout the whole genome with a bit higher frequency in GC-rich regions. By the DNA compositional analysis of genomic fragments of *T. scotoductus* SA-01 flanking the breakpoints it was found that a ‘GCGCGC’ motif was almost 4 folds more frequent in 40 base pairs upstream and downstream of the breakpoints than in the whole genome of *T. scotoductus* SA-01 in general. These (GC)_n_ repeats may facilitate homologous recombination between different chromosomal regions. The frequency of the oligonucleotide ‘GCGCGC’ was counted in non-coding sequences of *T. scotoductus* SA-01 and two genomes of *T. thermophilus* (Table 1). The oligomer was twice as likely in non-coding regions and in total per genome it was twice as likely in *T. thermophilus* in comparison to *T. scotoductus*. This observation was consistent with the assumption that in *Thermus,* genomic rearrangements more frequently occur between genes and between operons making the functional disruptions rare. And indirectly, this finding supports the hypothesis that the increased frequency of rearrangements in extreme thermophiles of *T. thermophilus* was an adaptive mechanism as the natural selection favoured accumulation of poly-GC motifs in *T. thermophilus* genomes in comparison to *T. scotoductus* SA-01. As these motifs presumably are genome rearrangement recognition sites, their accumulation may supposedly have increased levels of rearrangements. Schwarzenlander *et al.* [8] also reported that thermophilic organisms were characterized by frequent genome rearrangements and an increased genomic plasticity, although no biological explanations of this phenomenon were proposed.

**Table 1:** **Frequencies of the oligomer GCGCGC in coding and non-coding sequences of three** ***Thermus*** **genomes**

	***T. thermophilus***HB8	***T. thermophilus***HB27	***T. scotoductus***SA-01
Coding sequences	2.1	2.2	1.0
Non-coding sequences	4.6	4.0	2.1

Comparative analysis of the frequency of oligonucleotide words per 10 kbp for three thermophilic genomes within breakpoint regions.

*Thermus/Meiothermus* genomes comprise chromosomes, megaplasmids and small plasmids. The number of plasmids per genome differs between strains. For example, *T. scotoductus* SA-01 comprises one chromosome and one small plasmid TSCp8 (CP001963). Two relative organisms *T. thermophilus* HB8 and HB27 possess additional large plasmids, but the chromosomes of these organisms are shorter than that in *T. scotoductus* SA-01. There is an additional small plasmid in *T. thermophilus* HB8 that resembles TSCp8, but share no homology [15]. For the majority of genes present in the *T. scotoductus* SA-01 chromosome their orthologous counterparts are found in *T. thermophilus* chromosomes and plasmids [15]. Functional analysis of genes located on the megaplasmids of *T. thermophilus* showed that they encoded several metabolic pathways, namely: coenzyme B12 synthesis and metabolism; adenosylcobalamin biosynthesis and adenosylcobalamin salvage pathways; dATP, dGTP and dUTP biosynthetic pathways; neurosporene and siroheme biosynthesis. Other genes encoded different metabolic enzymes: acyl-CoA dehydrogenases, isomerases, oxido-reductase, glucosidases, galactosidases and some others. All these genes are spread on the chromosome of *T. scotoductus* SA-01 that probably was the case with the common ancestor of *Thermus* species. Considering evolutionary benefits that lay behind the movement of genes from the chromosome to the plasmid, one obvious benefit may be that two or several smaller replicons are faster replicating and may promote the organism propagation. Another explanation may be that the rate of mutations is higher on plasmids than on the chromosome and the population gets enriched in more variants of genes located on the mega-plasmids than those genes located on the chromosome [34].

### Horizontal gene exchange

**Figure 7:**
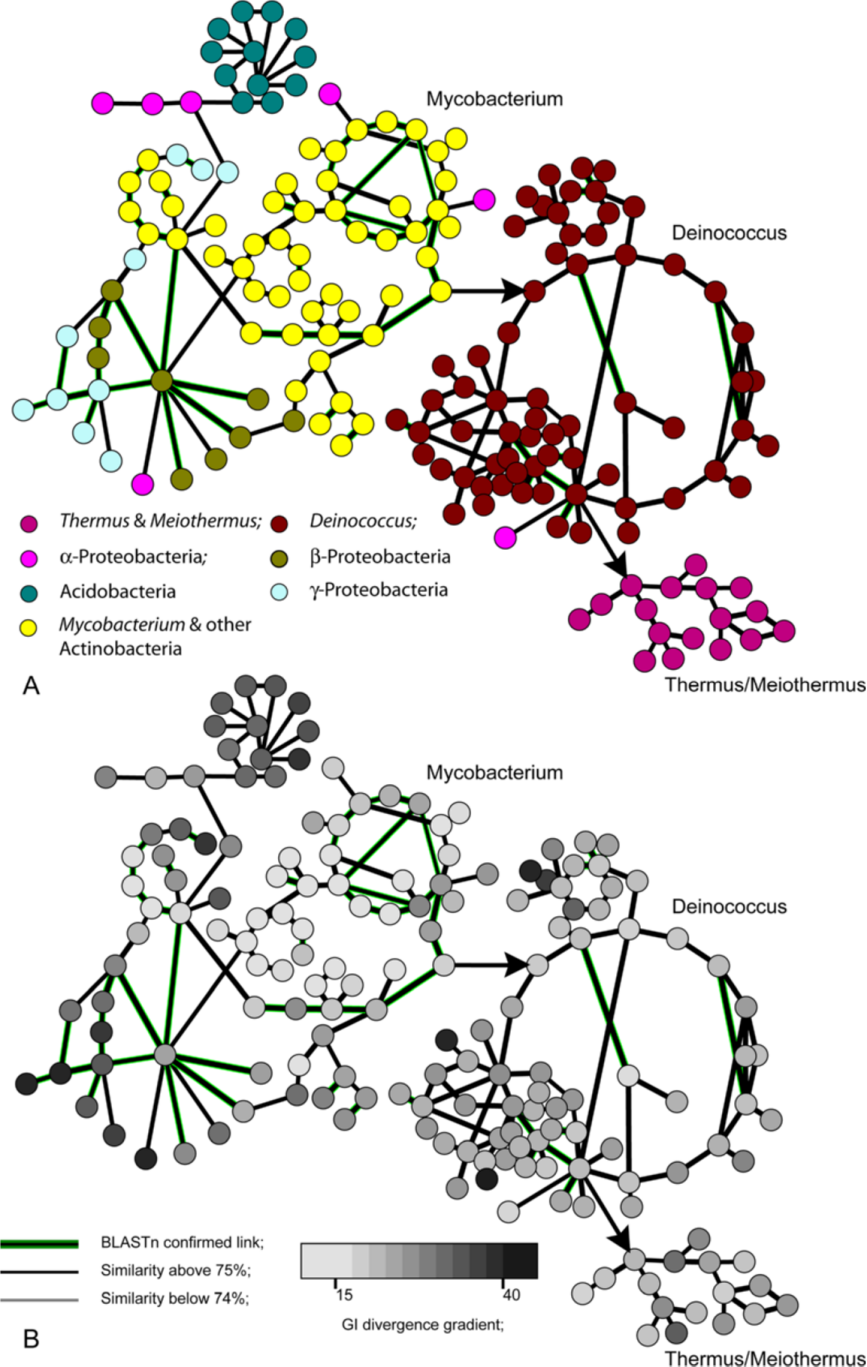
**Horizontal gene exchange between phyla and species. Every node represents one genomic island.** Links between nodes indicate a significant compositional similarity between sequences of these genomic islands. **A)** Colour code indicates phyla of host organisms where the genomic islands were detected. **B)** Grey scale indicates a relative time of insertion of genomic islands into chromosomes of host organisms. As lighter the colour, as elder the insertion.

Several horizontally transferred genomic islands (GIs) were identified in *Thermus* genomes and related species by SeqWord Sniffer program [15]. Genome atlases with the indicated positions of genomic islands are also available online [35]. A search through the database of genomic islands predicted in multiple completely sequenced bacterial genomes revealed a compositional similarity of genomic islands found in *Thermus* species to a broad group of mobile genetic elements discovered in *Deinococcus*, *Actinobacteria* and some other bacterial taxa (Figure 7A). After having genomic islands being transferred and incorporated into a chromosome, the mobile elements undergo DNA amelioration – a process that levels oligonucleotide usage (OU) patterns of the acquired genetic elements and host chromosomes [36]. Figure 7B shows the results of a stratigraphic analysis of genomic islands represented as linked nodes. The stratigraphic method calculates distances between oligonucleotide patterns of genomic islands and host chromosomes to determine the relative time of acquisition. In Figure 7B, the nodes which are depicted by a lighter colour show higher levels of compositional similarity to the host chromosomes than those depicted by a darker colour, which probably still resemble the composition of their donor genomes. Thus, the colours of nodes are related to the acquisition time. Recent acquisitions are distant from the hosts in terms of DNA composition; they therefore have a darker colour. A darker colour in this case means that these genomic islands have not lost their specific original composition yet. Overtime, these genomic islands have got affected in their new hosts by the directed mutational pressure (amelioration); they thus start to resemble the patterns of the host organisms. The lighter genomic islands in Figure 7B are ancient acquisitions, which have been in the host chromosomes much longer, hence the resemblance of the patterns.

The stratigraphic analysis demonstrated that prophages of mycobacteria were the most ancient genomic inserts. Later these genomic islands were acquired by *Deinococcus* (most likely *D. geothermalis* lineage) from where they were transmitted to *Thermus*, *Meiothermus* and *Deinococcus* species. But even in *Thermus* species, these genomic islands were relatively old as to compare to similar inserts in *β-* and *γ-*Proteobacteria (Figure 7B, see also Bezuidt *et al.* [37]). The majority of genes in genomic islands were annotated as conserved hypotheticals. It impeded inferring of the possible role that these genomic islands might play in the evolution of *Thermus*. Predicted functional genes in their majority are involved in cell wall polysaccharide biosynthesis that is in consistence with the previously reported observation that this category of enzymes was abundant in mobile genetic elements [37].

To identify possible horizontal gene transferring events through the sophisticated *Thermus* DNA uptake system [8, 14], phylogenetic trees were inferred by Neighbour-Joining for all 1,526 recognized groups of orthologous protein shared by 5 sampled *Thermus* and *Meiothermus* organisms. In every group, the gene tree was rooted to the sequence from *Meiothermus ruber* DSM 1279. Topology disagreements between gene trees were inspected by the Treedist program [23]. Mismatches between trees may be explained either by different rates of mutations in distant taxa, or by horizontal gene exchange. It was hypothesized that the differences in the rates of mutations most likely would affect lengths of branches in phylogenetic trees, while the horizontal gene transfer would cause predominantly topological changes. An exception may be if a gene in one of the organisms lost its functionality due to nonsense mutations or gene truncation that also may result in a tree topology alteration. To exclude these situations, only alignments with unambiguously aligned blocks selected by Gblocks comprising 75% or more of the initial alignment were studied.

Tree topology comparison revealed that the topologies of 1,384 gene trees were identical to the consensus tree. Eleven alternative tree topologies were found which were incongruent with the consensus tree and may be explained by horizontal gene transfer between these species or by acquisitions of genes from unknown sources. The topology in which *M. silvanus* was clustered together with two strains of *T. thermophilus* and *T. scotoductus* formed a group with *M. ruber* was second frequent one. Such topology may be explained either by the exchange of genes between *M. silvanus* and *T. thermophilus* lineages in any direction, or by exchange between *T. scotoductus* and *M. ruber* lineages, or by acquisition of diverse genes by *T. scotoductus* or *M. silvanus* from unknown lineages. To choose the most likely scenario, the following comparison was carried out. First, the average relative distances between 5 genomes were calculated based on 1,384 gene trees sharing the topology with the consensus tree. For normalization, the distances between corresponding nodes in a tree were divided by the total length of all branches of this tree. In trees with alternative topologies each genome was characterized by the amount of movement of the corresponding nodes in the tree relatively to other nodes as in equation 1:
1

where *S*_*ij*_ is the characteristic parameter calculated for the species *j* in the tree *i*; *dist*^*i*^_*jk*_ is the normalized distance between species *j* and *k* in the given tree *i*; and *dist*_*jk*_^*cons*^ is the distance between the same species in the consensus tree.

**Figure 8:**
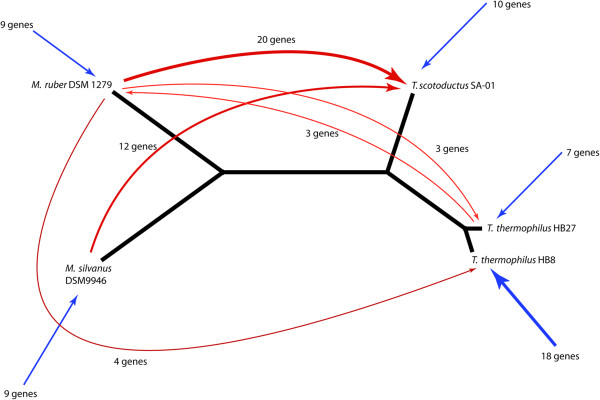
**Summary of donor-recipient gene exchange through horizontal gene transfer.**

The organism which gained the maximal *S*_*ij*_ in the tree was selected as the most likely recipient of horizontally transferred genes, and an organism which got closer to the recipient organism in the gene tree compared to the consensus tree was selected as a possible donor. If all distances from the recipient organism to others in the gene tree increased, it was assumed that the gene was acquired from an unknown source. Results of these calculations are summarized in Additional file 1: Table S1. It cannot be excluded in each particular case that an unexpected similarity between orthologous genes of two distant organisms may result from a genetic convergence rather than horizontal gene transfer. However, for the organisms possessing such an elaborated DNA uptake system as *Thermus* does, the hypothesis of the lateral gene exchange looks plausible. Even if not, all these genes have been horizontally acquired, this analysis may demonstrate which organisms tend more towards sharing the genetic material and which of them are inclined to be donors or recipients of DNA fragments. A summary of the most common donor-recipient links is shown in Figure 8. All identified mobile genes have been of chromosomal location in both donors (if known) and recipients. It was found that *T. scotoductus* more frequently acquired DNA fragments from *Meiothermus* than vice versa. It is quite possible that the relatively mildly thermophilic *Meiothermus* and *T. scotoductus* share their inhabitancy with each other more frequently than with the extremely thermophilic *T. thermophilus*. The latter organisms also made use of foreign DNA, but mostly from unknown sources. The capability of *Meiothermus* to uptake DNA fragments is noticeably weaker than that of *Thermus*. The genes which were possibly acquired by DNA transformation encoded for ribosomal proteins, enzymes of amino acid biosynthesis and some metabolic pathways. In contrast to genomic islands, which may bear genetic clusters encoding whole pathways, short DNA fragments comprise only one or few genes and usually replace homologous genes. Thus the conserved genes involved in the basic metabolic functions stand a better chance to get used in a new host and to persist over generations. Replacement of own genes by alternative foreign variants may be advantageous for the fine-tuning and timing of biological processes and protein-protein interactions to fit to specific environmental conditions.

## Conclusion

Bacteria of the *Thermus* genus, among which there are many industrially valuable strains, are known by their ability to acquire naked DNA fragments from the environment by using a specialized uptake system [8, 14]. Comparison of recently sequenced genomes showed a huge number of rearrangements of chromosomal loci even between closely related organisms [15] (Figure 3). Several alternative hypotheses were formulated at the beginning of this work such as that the frequent rearrangement may be caused by acquired pro-phages and other genomic islands, or they are controlled by some yet unknown internal mechanisms. It was unclear whether these rearrangements occur in a random manner, or they are controlled by some positive selection mechanisms, and if so, whether these forces related to the adaptive evolution of these bacteria towards survival in harsher environments?

It was found that the global genome rearrangements appeared to play an important role in this process. Whole operons and metabolic pathways were relocated in *T. thermophilus* onto the mega-plasmids. Probably, mega-plasmids are the places where the evolutionary processes are speeded up. This observation is in line with the definition of chromids – bacterial megaplasmids distinguishable from both bacterial chromosomes and plasmids [38]. In contrast to plasmids, the chromids carry core metabolic genes but they have plasmid replication system that usually is less reliable than the chromosomal one. According to Harrison *et al.* [38] the chromids are particularly rich in genus specific genes and appear at the origin of new genus evolution. This hypothesis is supported by the current observation that the *T. thermophilus* strains may benefit from the transfer of the evolutionary modifying genes onto the plasmid to achieve a higher level of genetic plasticity.

Even on the chromosomes of different *Thermus* and *Meiothermus* organisms their genes were significantly re-shuffled. By confronting evolutionary distances between the strains with the amounts of relocations of genomic fragments it was found that the rate of rearrangements is a bit higher in *Thermus* extreme thermophiles. The increased rate of genomic rearrangements led to some level of disintegration of functional operons in *Thermus*/*Meiothermus* that may be considered either as an effect of persistent environmental temperature stresses or as an adaptation process to fit better to extreme environmental conditions by splitting operons to smaller independent regulons. The observed marginal disintegration of operons may be a price that bacteria paid for the development of new more effective metabolic and regulatory pathways. In spite of a huge number of relocations, the functional disintegration of the metabolic network remained marginal as whole operons were more likely to be relocated than single genes or their parts [39] either because the latter events would be eliminated from the population by the natural selection, or because of a higher occurrence of rearrangement recognition sites between genes and operons. We observed that the genomic DNA composition might influence the rate of rearrangements. Rearrangement breakpoints were more frequent in GC-rich regions enriched with oligomers of specific types, which were rare in coding sequences. Interestingly, the frequency of these oligomers in non-coding genomic regions of *T. thermophilus* doubled in comparison to *T. scotoductus* that may explain the observed increase in rates of rearrangements in these organisms and indirectly it contributes to the hypothesis that the rate of genomic rearrangements is guided by the DNA composition and is an adaptive evolutionary process.

Another important factor of genome evolution is horizontal gene transfer that occurs through three different mechanisms: transformation, conjugation and transduction [40]. Large genomic islands found in *Thermus* organisms were predominantly old prophages similar to those in *Deinococcus* genomes. As they comprised mostly hypothetical genes, it was difficult to judge the role they possibly played in *Thermus* evolution. More intensive gene exchange between these micro-organisms occurred through transformation: a mechanism that is mediated by the uptake of DNA fragments from the environment. In *Thermus,* the DNA transformation is facilitated by availability of the unique DNA uptake system [8]. Genes, which were likely to be acquired horizontally, have been identified in this study by topological incongruence of gene trees compared to the consensus species tree. It was found that the gene acquisition by transformation is more frequent in *Thermus* rather than *Meiothermus* organisms but latter ones frequently are donors of genes for *T. scotoductus. T. thermophilus* strains also acquired DNA from the environment, but mostly from unknown donor organisms. This difference in horizontal gene acquisition between *T. scotoductus* and *T. thermophilus* may reflect either the specificity of their DNA uptake systems, or habitat specificity.

It was found that the extremely frequent genomic rearrangements between chromosomal and plasmid loci in *Thermus* genomes are moderated by internal mechanisms, which very likely contribute to the adaptive evolution of these bacteria. Whole operons more often are transferred as entities, thus the rearrangements usually do not disrupt syntenies of functionally related genes. We did not find any correlation between the rate of rearrangements and acquisitions of horizontally transferred genomic islands, but an increasing trend was observed in rearrangement frequencies in extreme thermophiles. Gene exchange by transformation were found to occur more frequently between thermophilic *T. scotoductus* and *Meiothermus* rather than between the extreme thermophiles. It may be explained either by the sharing of common habitats with moderate thermophiles, or by the fact that naked DNA fragments degrade much faster at extremely high temperature environments.

## Electronic supplementary material

Additional file 1: **Tree topologies for orthologous genes.** (XLSX 5 MB)
